# HIV proviral DNA integration can drive T cell growth ex vivo

**DOI:** 10.1073/pnas.2013194117

**Published:** 2020-12-14

**Authors:** John K. Yoon, Joseph R. Holloway, Daria W. Wells, Machika Kaku, David Jetton, Rebecca Brown, John M. Coffin

**Affiliations:** ^a^Program in Immunology, Graduate School of Biomedical Sciences, Tufts University, Boston, MA 02111;; ^b^Program in Pharmacology and Experimental Therapeutics, Graduate School of Biomedical Sciences, Tufts University, Boston, MA 02111;; ^c^Cancer Research Technology Program, Leidos Biomedical Research, Inc., Frederick, MD 21704;; ^d^Program in Genetics, Graduate School of Biomedical Sciences, Tufts University, Boston, MA 02111;; ^e^Department of Molecular Biology and Microbiology, Tufts University, Boston, MA 02111

**Keywords:** STAT3, HIV persistence, AIDS lymphoma

## Abstract

In vivo clonal expansion of HIV-infected T cells is an important mechanism of viral persistence. In some cases, clonal expansion is driven by HIV proviral DNA integrated into one of a handful of genes. To investigate this phenomenon in vitro, we infected primary CD4+ T cells with an HIV construct expressing GFP and, after nearly 2 mo of culture and multiple rounds of activation, analyzed the resulting integration site distribution. In each of three replicates from each of two donors, we detected large clusters of integration sites with multiple breakpoints, implying clonal selection. These clusters all mapped to a narrow region within the *STAT3* gene. The presence of hybrid transcripts splicing HIV to *STAT3* sequences supports a model of LTR-driven *STAT3* overexpression as a driver of preferential growth. Thus, HIV integration patterns linked to selective T cell outgrowth can be reproduced in cell culture. The single report of an HIV provirus in a case of AIDS-associated B-cell lymphoma with an HIV provirus in the same part of *STAT3* also has implications for HIV-induced malignancy.

Integration site analysis (ISA) of cells from HIV-infected donors has revealed large clusters of proviruses in the same orientation as transcription in a few genes related to T cell growth and survival, imparting long-term proliferative or survival benefits to the infected host cell (1, 2). Expanded cell clones have since been shown to harbor inducible replication competent virus (3). The study described in this report was designed to develop a system to study HIV integration-mediated selection in cell culture.

## Results

**Fig. 1.**
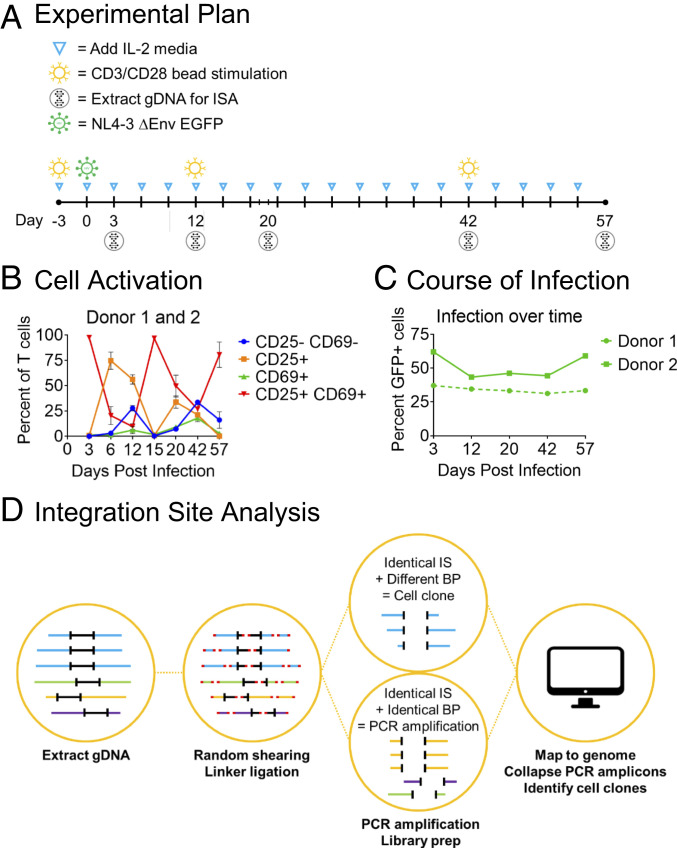
Experimental approach. (*A*) Timeline of CD4+ T cell culture, activation, infection, and DNA extraction for ISA. (*B*) Activation of T cells as measured by CD25/CD69 expression over the course of the experiment. (*C*) GFP expression during the study. (*D*) Overview of the ISA.

**Fig. 2.**
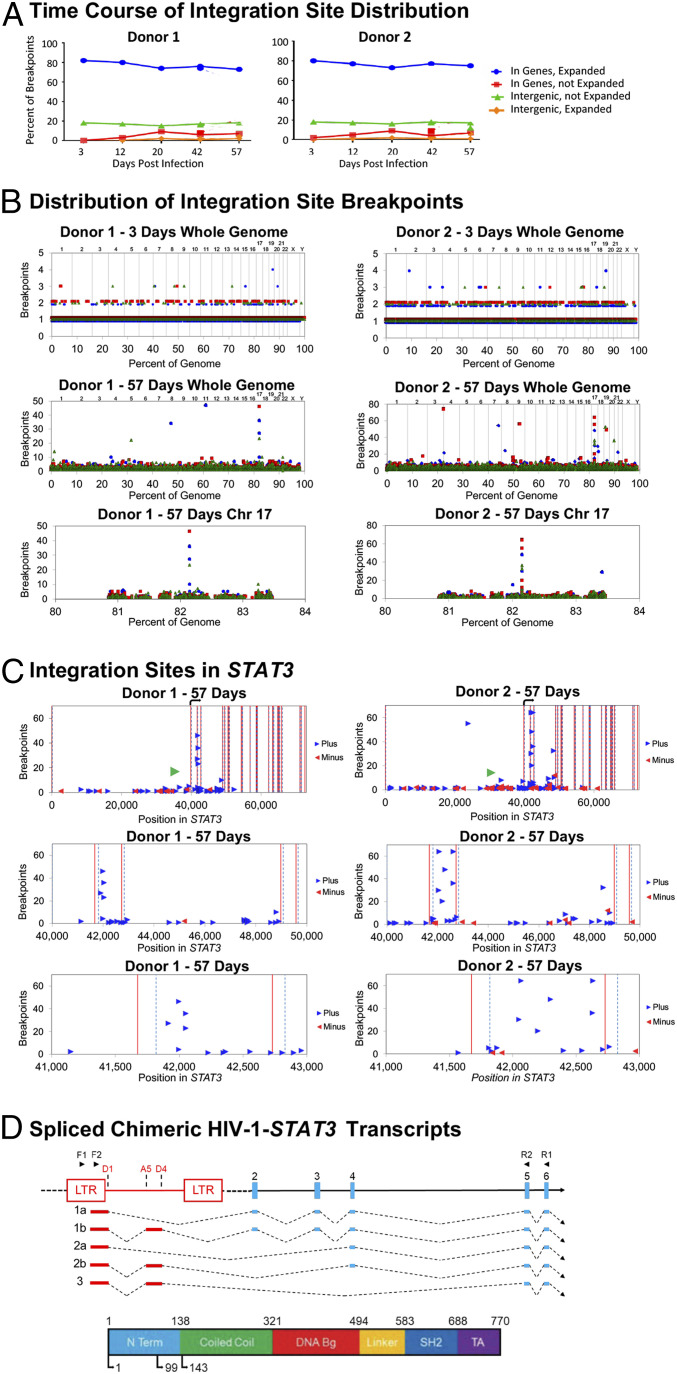
Clonal expansion of cells with proviruses in *STAT3*. (*A*) Time course of Integration site distribution. (*B*) Day 3 (*Top*) and day 57 (*Midddle*) integration site distribution and breakpoint totals across the entire genome and chromosome 17 (*Bottom*). Locations are percent of the total genome. Chromosome numbers are above each plot. (*C*) Day 57 integration site totals in *STAT3*. *Top* shows the entire gene; *Middle* and *Bottom* show successive amplification of the integration cluster. Lines represent intron boundaries: blue dotted lines, splice donor; red solid lines, acceptor. Transcriptional orientation is depicted left-to-right. Translation start site is indicated by the black arrows. The position of an HIV-1 provirus identified in a B cell lymphoma (10) is shown by the green arrow. (*D*) Structures of HIV–*STAT3* transcripts detected using RT-PCR (nested primer sets shown as black arrows). Start sites in the linear STAT protein structure 3 (reprinted from ref. 8, which is licensed under CC BY 4.0) at the bottom show truncations predicted from integration in introns 2 or 3 and 4.

We infected CD3/CD28-activated human primary naïve CD4+ T cells (Fig. 1*A*) from two donors in triplicate with a VSV-G pseudotyped HIV-1 vector, with GFP in place of *env,* at *ca.* 1 infectious unit per cell. Cultures were grown for ∼2 mo in the presence of IL-2 with periodic CD3/CD28 reactivation, while monitoring for activation markers (Fig. 1*B*) and frequency of infection (Fig. 1*C*). The frequency and distribution of proviral DNA integration into genomic DNA was assessed by the linker-mediated PCR integration site assay (Fig. 1*D*), incorporating a shearing step (2, 4) to assess the relative frequency of descendants of single infected cells. In all, 202,179 and 524,691 total integration sites were mapped from donors 1 and 2, respectively (Fig. 2*A*), out of which 165,190 and 464,487 represented unique events (Dataset S1). HIV proviral integration was found in 10,079 and 11,993 genes for donors 1 and 2, reflecting robust coverage across the genome.

As expected (5), genes were preferred for integration, and there was modest clonal expansion (more than two breakpoints per site) during the course of the experiment, with little indication of HIV-influenced expansion (Fig. 2*A*). At day 57, most integration sites were present in fewer than 10 breakpoints per site and were evenly distributed along all chromosomes (Fig. 2 *B*, *Middle*).

Most notably, however, dense clusters of integration sites were observed in cultures from both donors and all replicates on day 57 after the third reactivation (Fig. 2 *B*, *Middle*). These clusters mapped to a small region of the *STAT3* gene (Fig. 2*C*), with high levels of clonal expansion of cells containing proviruses within the first three introns in both donor sets (Fig. 2*C* and Dataset S2). As has been described previously for selected integrations (2), the highly expanded integration sites in *STAT3* were (with one exception) in the same transcriptional orientation as the gene, with all but two of them in a 10-kb region, mostly in intron 3. There was also a large expanded clone with a provirus upstream of the first coding exon in donor 2. The probability of these events occurring randomly is extremely low (*P* = 3.5 × 10^−10^).

In the extensively studied cases of oncogenic gene modification by oncogenic retroviruses (6), proviral insertion enhances overall transcription levels and can alter the structure of the protein product. In the present study, we detected long terminal repeat (LTR) *STAT3* spliced fusion transcripts in donors 1 and 2, in the day 57 samples (Fig. 2*C*). Most of the chimeric transcripts detected were predicted to result in proteins containing either a partial or complete deletion of the N-terminal domain (NTD) of *STAT3* (Fig. 2*C*). It is likely that enhanced expression of proteins encoding either full-length or partially truncated STAT3 protein imparted a selective advantage to cells.

We estimate that cells with proviruses in the selected region of *STAT3* increased about 100-fold in relative frequency over the course of the study, to about 0.3% of the population at day 57. From the relative breakpoint counts in the selected region of *STAT3* at day 57, as compared to day 42, and assuming a doubling time of about 1 d, this increase corresponds to an average growth advantage of about 18% per day (Dataset S2).

## Discussion

The STAT3 protein has a role in the expression of a variety of genes in response to cell stimuli, including in cell division (7). As such, constitutive expression and phosphorylation of STAT3 can play a role in deregulated growth and oncogenesis (7). Our results show that modification of STAT3 expression by HIV-1 proviral integration can promote cell overgrowth ex vivo, and suggest that the STAT3 NTD is not crucial for its proliferative promoting functions, in line with a previous result (8). The NTD is part of the dimerization surface in STAT3 and is important for efficient nuclear accumulation, DNA binding, and regulation of gene expression (8), including the regulation of proapoptotic gene expression in cancer cells (9). Additional studies looking into the impact of overexpressing NTD truncated STAT3 proteins and their effect on T cell growth and response to cytokine stimulation will help clarify the role of the STAT3 NTD.

HIV integration into *STAT3* has previously been linked, in a case report, to the formation of B cell lymphoma (10), in which a defective provirus integrated upstream of the first *STAT3* coding exon (Fig. 2 *C*, *Top*, green triangle). Although this is a single case, these data suggest that up-regulation of a protooncogene by HIV-1 insertional mutagenesis may have resulted in the development of a lymphoma, likely involving a mechanism similar to what we report here. Although HIV primarily infects CD4+ T cells, low-level infection of other cell types, including B cells, has been reported (11). Unlike CD4+ T cells, such cells, if transformed by proviral integration, would not be good targets for HIV superinfection and killing, allowing even rare infected cells to grow into a tumor. Further investigation of the implied direct role for HIV integration in AIDS lymphomas and other malignancies is obviously warranted.

While sporadic amplification of cells with integration sites in other genes was present (Dataset S2), likely reflecting a second provirus in some cells, there was no evidence of expansion linked to *BACH2*, *MKL2*, *STAT5B*, or any other gene, perhaps because selection for integration in these genes reflects a specific in vivo effect, such as suppression of cell death, rather than promotion of growth. Although insertional mutagenesis leading to cellular outgrowth, in the form of cancer, is well documented for other retroviruses (6), there are only a handful of reports describing HIV integration associated with cellular expansion (12–14). Indeed, it has been widely assumed that HIV, or HIV (“lentiviral”)-based vectors, cannot directly cause cancer because of its target cell and integration site specificity.

Although previous reports found evidence for HIV integration-mediated transformation of cells infected with Epstein−Barr virus (15), our experimental system should prove useful for studying the mechanism by which HIV integration can affect the growth properties of the normal cells they infect in vivo. It may also shed light on the mechanism of those AIDS-related malignancies for which no other infectious cause has been associated. Extending this study beyond the two donors analyzed will also be important to assess the generality of the phenomenon we have reported here.

## Materials and Methods

Naïve CD4+ T cells from two normal donors were obtained from Stemcell Technologies and infected with NL4-3 ΔEnv EGFP a VSV-G pseudotyped HIV-1 vector, with GFP in place of *env.* Cultures were maintained in triplicate in IL-2−containing medium, splitting as necessary (Fig. 1 *A*–*C*), and reactivated with CD3/CD28 stimulating beads at weeks 2 and 6. Longitudinal samples were harvested for ISA (Fig. 1*D*).

DNA was sheared by sonication, and subjected to linker-mediated PCR and paired-end sequencing using an Illumina HiSeq platform. Results were analyzed according to established ISA workflows (2, 4).

Total RNA was extracted, DNase treated, and reverse-transcribed into complementary DNA. Amplification of chimeric HIV–STAT3 transcripts was obtained by two sequential nested PCR reactions using primer sets complementary to the HIV LTR and *STAT3* exon 6/5. Amplified bands were cloned and Sanger sequenced. Sequences of the products obtained are shown schematically in Fig. 2*D* and have been deposited in GenBank under accession numbers MW323052–MW323066.

## Supplementary Material

Supplementary File

Supplementary File

Supplementary File

## Data Availability

Study data are included in the article and *SI Appendix*. All integration site data can be found in the Retrovirus Integration Database (https://rid.ncifcrf.gov/) and accessed using the PubMed ID of this paper. Sequences of the chimeric RNAs shown in Fig. 2*C* have been deposited in GenBank under accession numbers MW323052–MW323066.
