# Supplementary material for: HIV proviral DNA integration can drive T cell growth ex vivo
Source: Proc Natl Acad Sci U S A. 2020 Dec 14;117(52):32880–2. doi: 10.1073/pnas.2013194117 (PMC7777207; doi:10.1073/pnas.2013194117)
Supplement: Supplementary File [file pnas.2013194117.sapp.pdf]

Supplemental Datasets

Supplemental Dataset S1. The numbers of integration sites obtained for each sample at each time point. The file contains the total number of breakpoints seen in each of the three replicates from 2 donors at each of the 5 time points. “Total” sites include all breakpoints detected for each site; “unique” sites are recorded after collapsing the data to one breakpoint per site.

Supplemental Dataset S2. Expanded integration sites. The Microsoft Excel worksheet shows, for each donor, left to right, the day of sampling, the replicate culture number, the integration site (chromosome and nucleotide position of the base immediately 3’ to the provirus), the gene where integration occurred, the orientation of the provirus relative to the chromosome sequence, and, for each sampled day of the study, the number of sequences with the same integration site and different breakpoint in the flanking chromosomal DNA. All sites with 10 or more breakpoints (out of 726,870 total) found in the entire experiment at any of the 5 time points are shown. Independent integration sites in *STAT3* are highlighted. Note also that all proviruses but one in STAT3 with >10 breakpoints are in the (-) orientation, the same as *STAT3.* The results are summarized in the table to the right. Note that the expansion of the *STAT3* clones, collectively, amounts to an increase of > 10-fold relative to the total, consistent with a relative growth rate of 1.18 between the last two timepoints sampled, assuming a doubling time of 1 day.
